# Status epilepticus and early development: Neuronal injury, neurodegeneration, and their consequences

**DOI:** 10.1002/epi4.12601

**Published:** 2022-05-04

**Authors:** Kerry Thompson

**Affiliations:** ^1^ Occidental College Los Angeles CA USA

**Keywords:** development, neurodegeneration, seizures, status epilepticus

## Abstract

Evidence showing that the immature brain is vulnerable to seizure‐induced damage has been accumulating for decades. Clinical data have always suggested that some early‐life seizures are associated with negative sequelae, but clinical observations are frequently obscured by multiple uncontrolled contributing factors and can rarely establish causality. Determining with certainty that seizures, per se, can cause neuronal death and can irreversibly disrupt critical developmental processes, required the development of suitable model systems. Several experimental seizure models clearly show that the immature brain can sustain neuronal injury as a result of uncontrolled seizure activity and that even in the absence of observable neuronal death, the developing brain is selectively vulnerable to interruptions of required growth programs. Severe early‐life seizures inhibit DNA, RNA, and protein synthesis, and they can reduce the accumulation of myelin and synaptic markers in the developing nervous system, leading to functional delays in development. Depending on the seizure pathway involved, and the developmental period under study, classic neurodegeneration, excitotoxicity, and apoptosis can result in permanent damage to critical neural networks in the temporal lobe and in many other brain regions. This conclusion is further supported by recent clinical studies showing that prolonged febrile status epilepticus can lead to hippocampal injury, which evolves into hippocampal atrophy and hippocampal sclerosis. A growing body of experimental data demonstrates that the metabolic compromise and cellular loss produced by seizures during critical phases of brain development negatively affect later hippocampal physiology including learning and memory functions in maturity.

## 1 INTRODUCTION

**Key points**

- Several experimental seizure models clearly show that the immature brain can sustain neuronal injury as a result of uncontrolled seizure activity.
- Even in the absence of observable neuronal death, the developing brain is vulnerable to interruptions of required growth programs.
- Neurodegeneration, excitotoxicity, and apoptosis can cause permanent damage to critical neural networks in the developing temporal lobe.
- Metabolic compromise and cellular loss produced by developmental seizures negatively affect later hippocampal physiology.

A long‐standing debate over the impact of severe seizures in the developing nervous system may finally be closing in on consensus.^1^, ^2^, ^3^, ^4^, ^5^, ^6^ Pioneering clinical studies suggested^7^, ^8^, ^9^ and seminal basic developmental studies confirmed^10^, ^11^, ^12^, ^13^, ^14^ that severe early‐life seizures can cause significant cellular dropout and lasting developmental delays. Controversy arose when seizure models that reliably produced irreversible neuronal damage in mature animals,^15^, ^16^ failed to produce the same behavioral and histopathological outcomes in immature animals. Some of these important basic studies,^17^, ^18^, ^19^ as well as some clinical observations^20^ of early‐life seizures, suggested that the immature brain was resistant to seizure‐induced damage. For example, compared with adults, kainic acid seizures in young animals did not clearly show comparable levels of neuronal loss,^19^ although high mortality in the young mandated the use of a much lower dose of kainic acid,^21^ so that it was difficult to be sure that seizures were of comparable severity. These questions were addressed with a number of studies that established that when seizure‐associated repetitive firing of specific neuronal populations is sufficiently intense and prolonged, it can cause neuronal injury and death.^22^, ^23^ Later studies^24^ showed that neuronal vulnerability to seizure‐induced injury and/or death is highly dependent on circuit anatomy, age and brain developmental stage, and seizure type.

## 2 ANIMAL MODELS SHOW THAT NEURONAL LOSSES RESULT FROM SEVERE SEIZURES IN THE IMMATURE BRAIN

The development of new seizure models specifically in young animals revealed important information. The major point is that the immature brain can, and does, sustain damage in response to severe seizures that model status epilepticus (SE).^6^, ^22^, ^23^, ^24^, ^25^, ^26^ Seizure‐induced neuronal damage was demonstrated by modifying seizure models developed in mature animals and by the creation of new models. The addition of lithium (Li) pretreatment 24 h prior to pilocarpine injection allows a lower dose of the convulsant to induce SE.^27^ A lower dose of pilocarpine (PC) reduces many of the systemic side effects of higher doses and reduces the high mortality rates reported in young rats.^18^ LiPC SE was shown to produce a massive loss of many hippocampal and neuronal populations in immature (postnatal day [PND] 10) rabbits^23^ and, to a lesser degree, temporal lobe damage and synaptic remodeling in rats between 2 and 3 weeks of age.^24^ New models of febrile seizures, the most common seizure type in the young, also demonstrated damage in the immature brain. Toth et al. (1998)^28^ showed that hyperthermia‐induced seizures produce profound damage in the hippocampus and the amygdala in PND 10 rats. Significant temporal lobe damage was revealed with silver staining 24 h following the seizure.^28^ The combination of LiPC with hyperthermia in a more recent set of experiments allowed a dissection of the effect of SE alone and SE in the context of high brain temperature.^25^ It was shown that lesions of the hippocampus were produced in both of these conditions but involvement of the amygdala was more likely with the contribution of hyperthermia.

**FIGURE 1:**
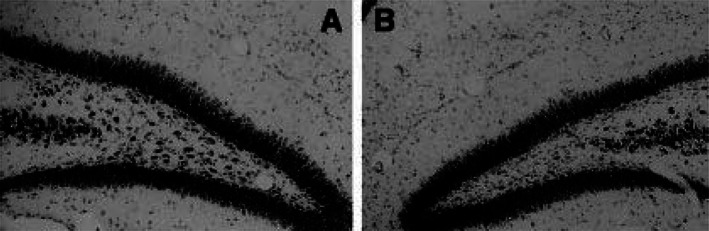
Loss of large (>15 μm) hilar neurons after perforant path stimulation in the young rat. In the areas of most severe cell loss, cell counts in sections stained with cresyl violet revealed significant reductions in the large hilar interneurons in the stimulated hippocampus (B), compared with the unstimulated hippocampus (A). (Image taken from reference #22, figure 6 Elsevier science ltd. with permission)

**FIGURE 2:**
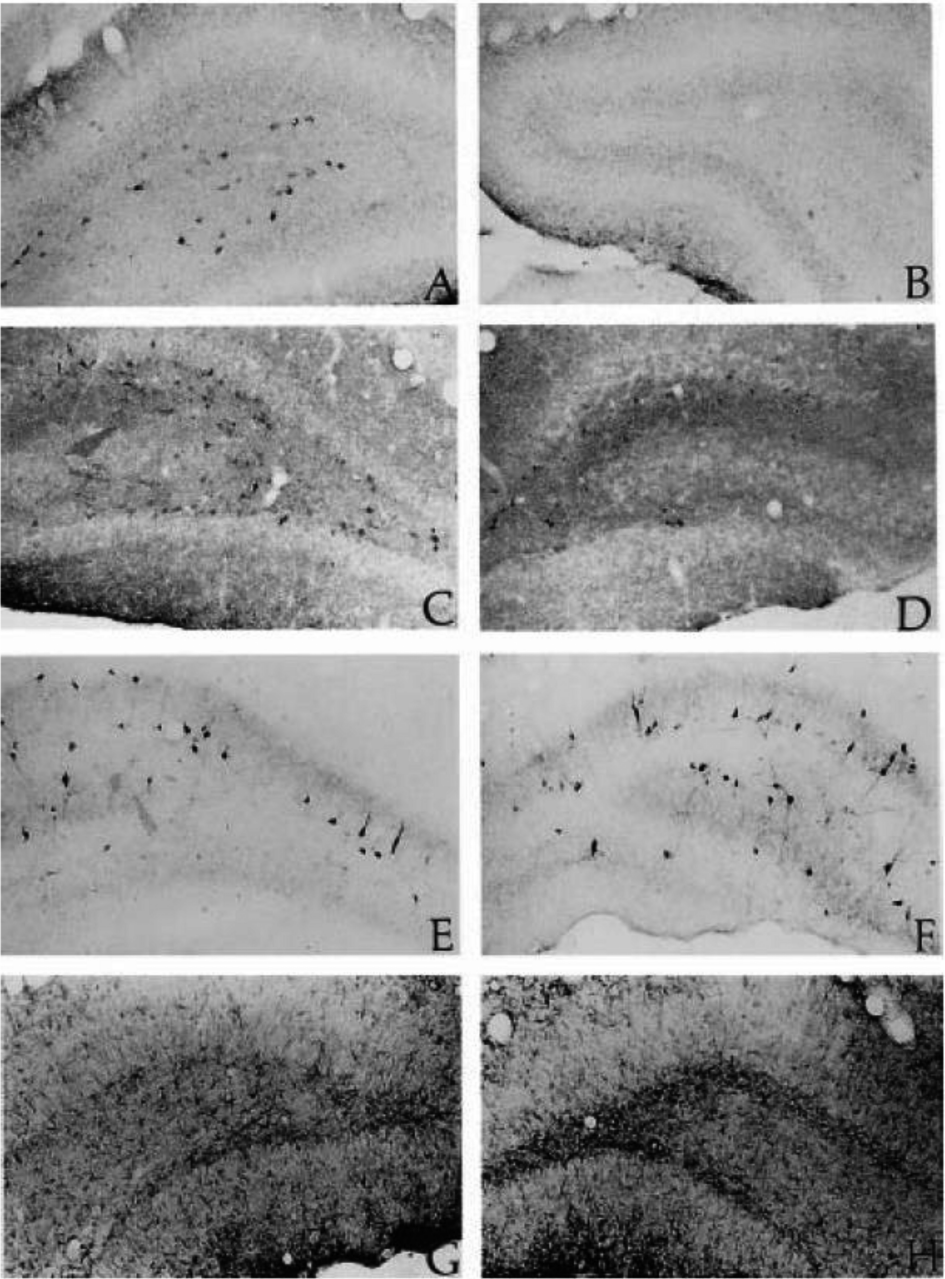
Immunohistochemical staining on month after perforant path stimulation in young rats. Indirect immunohistochemical analysis showed significant losses in somatostatin and neuropeptide Y‐immunoreactive cell in the stimulated hilus (B,D) compared with the unstimulated hilus (A,C). No differences were seen in the parvalbumin staining patterns between the stimulated (F) and unstimulated (E) sides. GFAP staining showed increased staining in the stimulated hilus (H) compared with the unstimulated hilus (G). (Image taken from reference #22, figure 7 Elsevier science ltd. with permission)

Another major point emerged with the development of new seizure models in the young. The unique physiology of the developing nervous system has to be considered when both developing, and when evaluating outcomes, in these models. An example of this point was revealed with the development of the perforant path stimulation (PPS) model in PND 14 rats.^22^ This model was based on the elegant demonstration that unilateral seizure‐like stimulation of the perforant path, under urethane anesthesia produced unilateral hippocampal lesions that included the loss of key GABAergic interneurons and physiological inhibition.^29^ The lesion in the perforant path stimulation model resembles lesions described in resected tissue from patients with temporal lobe epilepsy.^30^ When it was discovered that urethane, like other sedative‐hypnotics, induced apoptosis in the young brain,^31^ stimulation without anesthesia was used. The animals were stimulated ~16 hours in the awake state using the same parameters that were later found to induce self‐sustaining (SS) SE in older animals.^32^ The young animals exhibited “wet dog shakes” and hindlimb scratching, but they never entered SSSE. Nonetheless, the stimulation caused acute injury and death of hilar interneurons on the stimulated side only (Figure 1), and a chronic loss of somatostatin‐immunoreactive hilar interneurons on that side when studied a month later (Figure 2). These lesions could not be due to systemic seizure‐induced changes, since they were only present on the side of stimulation.^22^ The “electroclinical dissociation” described specifically in the young^33^ was clearly evident in this model. The neuronal damage included an early dropout of TUNEL‐positive cells within the inner layer of the granule cells that has not been reported in the adult model. Additionally, there were unique inhibitory circuit responses following injections of the NMDA blocker MK‐801. Prior to seizure‐like stimulation, tests of hippocampal inhibition revealed a loss of GABA‐mediated paired‐pulse inhibition when NMDA channels were blocked.^34^ MK‐801 produced neuroprotection in the hippocampus but was associated with a concurrent spread of damage to the entorhinal, and retrosplenial, cortices. These are clear indications that the circuitry and physiology of the immature brain, although vulnerable to seizure‐induced damage like the adult, cannot be uncritically compared with the mature nervous system.

The idea that early‐life seizures, per se, are benign, will influence how aggressively they are treated,^35^ as clinicians look for the underlying pathology causing the seizures and treatment itself may come with some costs.^36^ Rapid and aggressive seizure suppression would be a more reasonable treatment strategy if prolonged, or recurrent, seizure activity caused significant cell loss,^37^ or if they interrupted critical developmental milestones.^13^ These alternatives hypotheses have been informed by a number of studies^10^, ^22^, ^23^, ^25^, ^38^ that establish a clear principle; the complex physiology of the developing peri‐ and neonatal central nervous system^33^ contains critical time windows of vulnerability to metabolic insults,^39^, ^40^, ^41^ including the disruptive pathophysiology of severe seizures.^5^, ^24^, ^26^, ^42^ Some early clinical observations^20^ of early‐life status epilepticus also suggested a relatively benign outcome, but more detailed studies of febrile status epilepticus showed that it is followed in a significant proportion of patients by hippocampal atrophy and hippocampal sclerosis on magnetic resonance imaging^1^, ^3^ and by other subtle anatomical and behavioral changes.^2^, ^43^ Severe epileptic encephalopathies like Dravet's syndrome produce profound behavioral deficits, but it is currently unknown whether uncontrolled seizures contribute to behavioral sequelae or not. Improvements in seizure control may answer those questions in the near future.

The negative impact of seizures can manifest on a spectrum ranging from acute detectable neuronal death (limbic and/or extralimbic)^6^, ^22^, ^23^, ^24^, ^44^, ^45^ to more insidious long‐term structural and functional deficits in key circuits of the CNS, without observable evidence of cell loss.^4^, ^5^, ^13^, ^46^ Predicting the potential negative impact of early seizures, and the biological substrates that govern it, will depend on a careful analysis of current models and the development of new models systems.^47^ Progress in the treatment of, and protection from, the damage caused by early‐life seizures, will require modeling that takes into consideration the developmental stage of the CNS at the time of the insult, and what vulnerabilities exist acutely to seizure discharge within a developing cell type, or within a developing circuit. Our increasing understanding of CNS development brings attention to critical processes that seizures are likely to disrupt. These highly regulated processes include the following: activity‐dependent gene expression, synapse formation and circuit retention, cell cycle control, and programmed cell death during critical periods of development. Experimental models need to consider and analyze how sustained pathological cell firing in the early postnatal period might translate into long‐term circuit alterations and functional deficits.

## 3 EVEN IN THE ABSENCE OF NEURONAL DEATH, SEIZURES MAY CAUSE LASTING SUBTLE STRUCTURAL AND/OR FUNCTIONAL DEFICITS

Metabolic insults during critical periods of development can cause irreversible damage to the developing nervous system. If key physiological processes in the developing brain are significantly perturbed at critical time‐dependent steps, both acute and long‐term negative consequences are the result. Normophysiologic conditions are required as the developing CNS rapidly progresses, step‐wise, through periods of neurogenesis, cell migration, neuronal differentiation, synapse and circuit formation and retention. Each area of the brain is associated with different developmental time windows for these critical periods.^48^ This principle was elegantly demonstrated in the development of the cat visual system by Hubel and Wiesel.^49^ In that system, proper synapse and circuit formation requires coordinated physiological levels of activity from both eyes in the first few weeks of life. If normal physiological activity is prevented for the entirety of that critical period, severe circuit alterations become permanent. The principle of physiologic disruptions during critical periods causing irreversible damage in the developing brain can be extended to hormonal insufficiency,^39^ severe malnutrition,^41^ and seizures.^14^ Importantly, catastrophic damage to the developing postnatal CNS does not always require prolonged or recurring periods of physiological compromise, and millions of cells can be deleted within hours of a single insult with temporal and anatomical dependency.^31^

CNS development progresses on a sliding temporal–spatial scale stretching from embryonic neural tube closure to the final pruning of the prefrontal cortex in the adult mammalian system. Studies, conducted primarily in rats, have shown that severe or repeated seizures can significantly affect brain growth processes that are occurring at scale in the perinatal period.^11^, ^12^, ^14^ This is a period where active cell‐cycle genes are still being expressed in the superficial layers of the cortex^50^ and when neurogenesis is robust in the developing dentate gyrus.^51^ These seizure‐related effects on growth wane as the CNS exits the “brain growth spurt” when critical periods of development begin to close.^14^ Wasterlain and Plum (1973) showed long ago that repeated electroconvulsive seizures and status epilepticus^14^ significantly reduce RNA and DNA synthesis and that the outcome is critically dependent on the timing of the insult.

The findings of the negative growth impact of early seizures during the brain growth spurt were validated with the use of chemically induced seizures models^12^ and dissociated from the contributing effects of malnutrition.^40^ Those early studies were designed to dissect negative growth effects that might interrupt development and the attainment of behavioral milestones (which they did^13^) from seizure‐induced cell death in the developing brain. More recent studies have demonstrated that perinatal seizures reduce neurogenesis and are associated with aberrant mossy fiber circuit development in the hippocampus.^4^, ^53^ Importantly, developmental seizure‐induced effects translate into long‐term learning and memory deficits^53^ and a sustained deficit in normal hippocampal place cell physiology.^54^

The clinical prognosis for children experiencing severe early‐life seizures includes cognitive delays^8^, ^55^, ^56^, ^57^ but there are also ample clinical data linking severe, or recurrent, childhood seizures with cell damage.^58^, ^59^, ^60^ Since the clinical picture can be obscured by multiple variables such as preexisting damage, or an underlying disease processes that can cause damage, animal models are essential to understand the histopathological outcomes of early‐life seizures.^47^, ^61^ As mentioned above, when seizure models that reliably produce damage in the mature brain were adapted to young animals, the results did not clearly show comparable levels of neuronal loss.^17^, ^18^, ^19^ These basic studies were influential, and so too were clinical reports that also appeared to support the concept that the immature brain might be resistant to seizure‐induced damage.^20^ However, the use of seizure models originally developed in the adult is tenuous, because of the differences between the neurobiology of the developing nervous system, compared to that of the mature nervous system.^33^

Attention to age‐specific factors underlying the pathogenic mechanisms and sequelae of neonatal seizures has been discussed in previous reports.^52^, ^53^, ^62^, ^63^, ^64^ The benefit of having multiple developmental seizure models is that many of the important questions can be addressed using a model‐and age‐specific approach.^62^, ^65^ Models of perinatal seizures that occur during periods of intense DNA synthesis are linked to reduced mitotic activity, but not conventional excitotoxic lesions.^12^, ^66^ In the first few days of postnatal life, the cortical development continues as neurons are gradually attaining their postmitotic state^50^ and hippocampal neurogenesis continues into adulthood.^51^ These models can be used to study the dysregulation of the cell cycle, which is critically dependent on depolarization states mediated by GABA and glutamate,^67^ ionic balance,^68^ and calcium regulation,^69^ which are all affected by seizures. The suppression of RNA and synaptic protein synthesis in these models^70^, ^71^ may be linked to circuit modifications and later cognitive deficits.^53^ Models that produce clear histopathological outcomes in the young can be used to develop neuroprotective strategies, but with the understanding that NMDA receptor blockers and GABAmimetics have the potential to induce damage in the developing brain^34^, ^72^ and in some cases may worsen outcomes.^73^ The late expression of chloride extruding transporters in development^74^ and the relatively late development of GABA receptors relative to certain classes of glutamate receptors^33^ help explain why the immature brain is seizure prone, but also provide therapeutic targets in developmental seizure models. Finally, the continued development of new models like the LiPC SE model plus hyperthermia will allow a greater understanding of specific vulnerabilities of neuronal populations at particular developmental stages and facilitate the development of rational neuroprotective strategies like brain cooling.^25^

The immature brain can be harmed by repeated and/or severe seizures.^37^, ^75^ Seizure‐induced death occurs in the developing brain as a result of intense neuronal firing in the absence of systemic changes.^22^, ^23^ Pathophysiological changes can occur in the presence or absence of observable damage and likely along occur along a continuum. These changes have been shown to have lasting effects on hippocampal physiology and behavior,^13^, ^76^ including negative impacts on measures of learning and memory.^4^, ^53^ It is clear that seizure‐induced damage or disruption of physiological neuronal activity during critical periods of development can have deleterious consequences that extend well into maturity.

I confirm that I have read the Journal's position on issues involved in ethical publication and affirm that this report is consistent with those guidelines.

## CONFLICTS OF INTEREST

I have no conflicts of interest to disclose.
